# The Effect of Canal Preparation with Four Different Rotary Systems on Formation of Dentinal Cracks: An In Vitro Evaluation

**DOI:** 10.22037/iej.v13i2.16416

**Published:** 2018

**Authors:** Elham Khoshbin, Zakiyeh Donyavi, Erfan Abbasi Atibeh, Ghodratollah Roshanaei, Faranak Amani

**Affiliations:** a *Department of Endodontic Dentistry, Dental School, Hamadan University of Medical Sciences, Hamadan, Iran; *; b *Department of Periodontology Dentistry, Dental School, Urmia University of Medical Sciences, Urmia, Iran; *; c * Modeling of Noncommunicable Diseases Research Center, Department of Biostatistics and Epidemiology, Public Health School, Hamadan University of Medical Sciences, Hamadan, Iran; *; d * Dental Center, Urmia Seyedoshohada Hospital, Urmia University of Medical Sciences, Urmia, Iran*

**Keywords:** Dentinal Crack, Endodontics, Root Canal Preparation, Rotary System

## Abstract

**Introduction:**

Endodontic rotary systems may result in dentinal cracks. They may propagate to vertical root fracture that compromises the outcome of endodontic treatment. This study aimed to compare Neolix and Reciproc (single-file systems), Mtwo and ProTaper (conventional rotary systems) in terms of dentinal crack formation in root canal walls.

**Methods and Materials:**

This *in vitro* study was conducted on 110 extracted human single-rooted teeth. The teeth were randomly divided into four experimental groups (*n*=25) for root canal preparation with Neolix, Reciproc, Mtwo and ProTaper systems and two control groups (*n*=5). The first control group underwent root canal instrumentation with hand files while the second control group received no preparation and was only irrigated. After instrumentation, root canals were horizontally sectioned at 3, 6 and 9 mm from the apex and inspected under a stereomicroscope under 12× magnification for detection of cracks. The data were analyzed using *Chi-square, *GEE test and Bonferroni tests (*P*<0.05).

**Results:**

No crack was found in the control groups. All rotary systems caused dentinal cracks. ProTaper, Reciproc, Mtwo and Neolix caused cracks in 92%, 80%, 68% and 48% of samples. ProTaper caused significantly more cracks than Neolix and Mtwo (*P*<0.05). No significant differences were noted between other groups (*P*>0.05).

**Conclusion:**

All rotary systems cause dentinal cracks and it is significantly different in apical, middle and coronal third of the root. Neolix appears to be a suitable alternative to other rotary systems since use of this single-file system saves time and cost and minimizes trauma to dentinal walls.

## Introduction

Vertical root fracture (VRF) is an important clinical problem, which compromises the outcome of endodontic treatment. Complete and incomplete dentinal cracks in the root canal wall may propagate and result in VRF [1, 2]. Several factors may play a role in formation of dentinal cracks in root canal walls such as high concentration of sodium hypochlorite (used for root canal irrigation), condensation during root canal filling (particularly lateral compaction), some root canal cleaning and shaping techniques and dentin dehydration [3, 4]. 

During root canal shaping, geometry of rotary systems, cutting blade design, taper of files and their composition all affect root dentin. These factors along with the diameter of prepared root canal may be responsible for dentinal crack formation and subsequent development of VRF [5, 6]. The main goal of chemical and mechanical root canal preparation is to eliminate microorganisms, pulpal tissue and debris from the root canal system and flare the root canal for adequate filling [7]. 

Chemical and mechanical preparation of the root canal system may traumatize the root dentin and result in dentinal crack formation or vertical root fracture, which decrease the long-term prognosis of endodontically treated teeth [6, 8]. 

In the recent years, advent of nickel titanium (NiTi) files and rotary systems revolutionized endodontic treatment. These instruments decrease the clinician’s fatigue and enable faster root canal treatment. Also, rotary instruments decrease the risk of procedural errors compared to hand files [9, 10].

To increase the efficacy of NiTi rotary instruments, advanced designs with non-cutting tips, radial land, different cross sectional designs, high torsional fracture strength and different tapers have been introduced. Most of these instruments have tapers in the range of 4 to 12%, which are greater than the ISO standard of 2%, and apply considerably high stress to root dentin [3], because root canal preparation with rotary instruments compared to hand files requires higher rotations of instruments inside the canal [4]. Due to the variability in types of rotary systems available in the market and limited information on the quality of new systems, assessment of the efficacy of these systems for root canal treatment is a priority. 

Today, ProTaper and Mtwo are used widely. ProTaper rotary system (Dentsply Maillefer, Ballaigues, Switzerland) has a convex triangular cross-section with variable taper [11]. It consists of SX (auxiliary shaping file, tip size 17) used for the coronal portion of the root canal, followed by S1 (tip size 20) in the coronal third and S2 (tip size 19) in the middle third; followed by F1 (20/0.07), F2 (25/0.08) and F3 (30/0.09) and F4 (40/0.06) finishing instruments [12]. Mtwo rotary system has S-shaped cross-section and a non-cutting tip. They have positive rake angle with two cutting edges [1, 13].

Recently, Neolix single-file rotary system was introduced to the market. Neoniti A1 (NEOLIX, Châtres-la-Forêt, France) has continuous rotating movement and is made up of special alloy that permits the file flexibility. This system is produced with three different sizes (20/0.08, 25/0.08 and 40/0.08) that are recommended to be used with speed of 300 to 500 rpm and torque limit of 1.5 N/cm. It has a non-uniform square- or rectangular-shaped cross-section along the blades, which confers optimal flexibility to the file. Also, in contrast to other NiTi files, Neolix file can be pre-curved. It has a non-cutting tip and provides easy and safe access to the apex. It enables efficient instrumentation of root canal with only one rotary NiTi file [14]. 

Reciproc (VDW, Munich, Germany) is another recently introduced single-file engine-driven system. The file is made of M-Wire NiTi alloy, which enhances flexibility while maintains cutting ability. Reciproc has S-shaped cross-section, a non-cutting tip and sharp cutting edges that shapes the canal by means of a reciprocal back-and-forward motion with a speed of 300 rpm (150 degrees counterclockwise and then 30 degrees clockwise). This single file system is available at three different sizes and tapers; R25 (25/0.08), R40 (40/0.06) and R50 (50/0.05) [12]. Due to the reciprocal motion, pattern of stress applied to the root canal walls is expected to be different from that of conventional rotary systems [8]. Considering the increasing use of NiTi rotary instruments and the adverse effects of cracks and root fracture on prognosis of endodontically treated teeth as well as limited studies on the possibility of dentinal crack formation by Neolix and Reciproc single-file systems, this study aimed to compare dentinal crack formation in root canal walls following instrumentation with Neolix as a single-file rotary system, Reciproc as a single-file reciprocating system, and Mtwo and ProTaper as conventional rotary systems.

## Materials and Methods

This *in vitro* experimental study was conducted on 110 freshly extracted single-rooted, single-canal human teeth with no apical root curvature. The teeth had been extracted due to orthodontic or periodontal reasons. The study protocol was approved in the ethics committee of Hamadan University of Medical Sciences (code: 9412257396). The teeth were randomly divided into four experimental groups (*n*=25) and two control groups (*n*=5). The teeth were stored in distilled water before and during the experiment [15]. The inclusion criteria were having a single root and a single straight canal with a closed apex, no root curvature, no cracks or fracture, no dental anomalies, absence of extensive caries or root resorption and no history of previous endodontic treatment. Open apex teeth and those with root curvature, anatomical anomalies or a fracture line were not included. Sample size was calculated to be a minimum of 25 teeth in each group using sample size calculation formula assuming 95% confidence interval of 0.975 and study power of 80%. 

The teeth were inspected under a loop with 2.5× magnification (Zinnor, Deck Inc., USA) to ensure that they met the inclusion criteria and were then stored in distilled water until use [13]. The teeth were also radiographed to ensure presence of a single root canal, and root width was measured in buccolingual and mesiodistal dimensions at 5 mm from the apex using software ruler to standardize the teeth in this regard and ensure their comparability [13]. The crowns were cut by a low speed handpiece under air and water spray such that the remaining root length was 16 mm. All roots were inspected under a stereomicroscope under 12× magnification and those with cracks were excluded and replaced with sound teeth. 

The teeth were then randomly divided into four experimental groups (*n*=25) of Reciproc, Neolix, Mtwo and ProTaper and two control groups (*n*=5). A #10 K-file was used to obtain patency and was introduced into the canal until its tip was visible at the apex. Working length was determined as such. Root canal preparation in the four groups was performed according to the manufacturers’ instructions by the same clinician as follows:

**Figure1:**
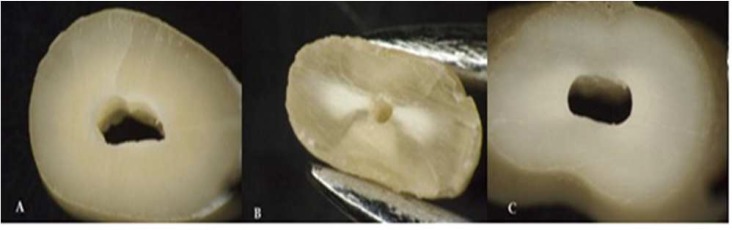
Samples instrumented with rotary systems under a stereomicroscope under 12× magnification; *A)* Presence of a complete crack; *B)* Presence of several cracks; *C)* Absence of crack

***Root canal preparation with ProTaper***

In this group, root canal preparation was done using crown-down technique according to the manufacturer’s instructions. The ProTaper system (Dentsply Maillefer, Ballaigues, Switzerland) was used Endo IT electromotor (VDW, Munich, Germany). Each file was used for preparation of four teeth only [16, 17].

***Root canal preparation with Mtwo***

In this group, root canal preparation was done using single length technique according to the manufacturer’s instructions. Mtwo rotary files (VDW, Munich, Germany) were using in the following sequence: 10/0.04, 15/0.05, 20/0.06, 25/0.06 by brushing movement and without application of pressure. The rotary files were mounted on and handled by Endo IT (VDW, Munich, German) with 280 rpm speed [18, 19].

***Root canal preparation with Reciproc***

First, a #20 K-file reached the working length and then #25 Reciproc file (VDW GmbH, Munich, Germany) was activated in reciprocating motion using VDW silver electric motor (VDW GmbH, Munich, Germany). According to the manufacturer’s instructions, activated file was gradually introduced into the canal with in and out pecking movement with 3 mm range and brushing motion. After three pecking movements, the file was removed and root canal was rinsed with 3 mL of saline. After three repetitions, a #10 K-file was used to ensure patency. This process was repeated until reaching the working length [15]. 

***Root canal preparation with Neolix***

Endo IT electromotor (VDW GmbH, Munich, Germany) was used to control speed and torque in this group. Neolix files (Neolix Xavier, Châtres-la-Forêt, France) were used at 300-500 rpm and 1.5 N/cm torque with pecking and brushing motions as recommended by the manufacturer. First, C1 file was used for flaring of the root canal orifice and removal of dentinal barriers with brushing motion (only in the coronal third). Next, #25 A1 file was passively used to prepare the middle and apical thirds of the canal. After three to four brushing movements, root canal was rinsed with saline and its patency was ensured using a #15 K-file. File with pecking motion was used to reach working length and complete shaping of root canal [14].

***Control groups***

In the first control group (*n*=5), root canals were prepared with hand files. First, a #10 K-file was used to obtain patency. After determining the working length, root canal preparation was done with watch winding and circumferential filing. Filing was done using #15 to #45 files (Kiyohara Industrial Park, Utsunomiya, Tochigi, Japan). Recapitulation was also performed and root canals were rinsed with 3 mL of 2.5% sodium hypochlorite (Kimia Tehran Acid, Tehran, Iran) in-between filing. In the second control group (*n*=5), no instrumentation was performed and the root canals were only irrigated with 2.5% sodium hypochlorite. 

After root canal preparation, the roots were horizontally sectioned at 3, 6 and 9 mm from the apex using a diamond disc. The sections were inspected under a stereomicroscope (Olympus Optical Co LTD, model:SZX/I/B200/, Tokyo, Japan) under 12× magnification (Figure 1). 

***Statistical*** ***analysis***

The data were analyzed using SPSS software (SPSS version 21, SPSS , Chicago, IL, USA) *via* *Chi*-square test, GEE test and Bonferroni test. Level of significance was set at 0.05. 

## Results

**Figure 2:**
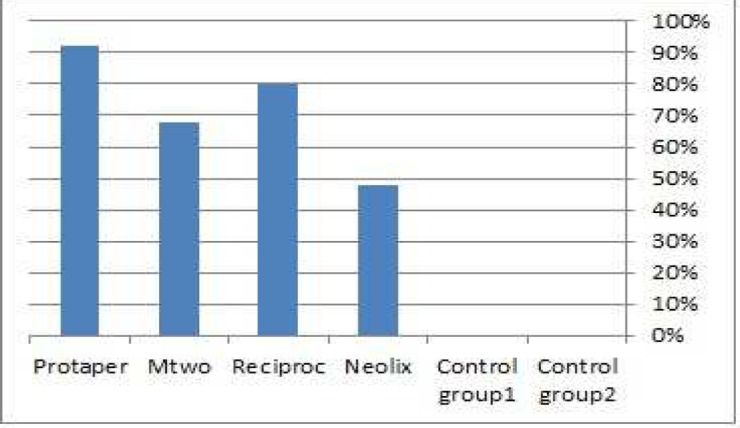
Percentage of dentinal cracks formation found in study groups

No crack was noted in the control groups. In the experimental groups, the highest and lowest number of cracks was respectively found in root canals prepared with ProTaper (92%) and Neolix (48%) (Figure 2). GEE test was used to compare experimental groups in terms of the frequency of cracks and the results showed a significant difference in this regard among groups (*P*=0.006). Bonferroni test was applied to multiple comparison between the experimental groups in terms of the frequency of cracks, which showed the statistically significant difference only between Neolix/ProTaper (*P*=0.023) and Mtwo/ProTaper (*P*=0.049).

**Figure 3:**
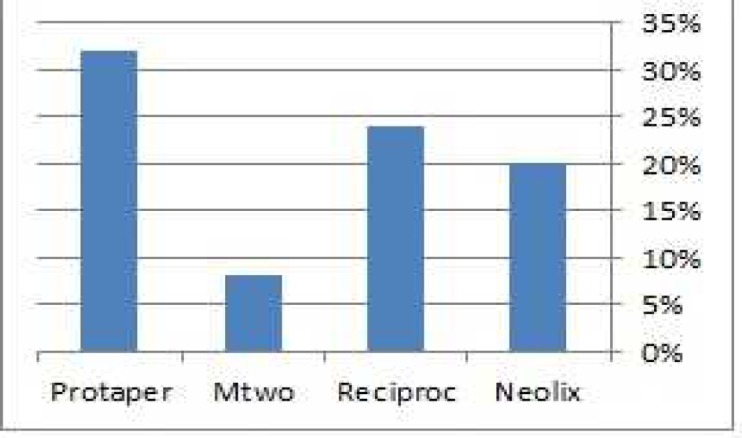
Percentage of dentinal cracks formation found in experimental groups, in level 1

**Figure 4:**
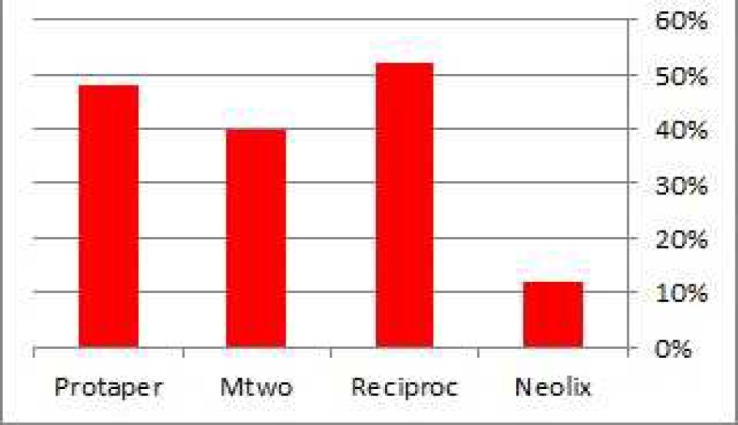
Percentage of dentinal cracks formation found in experimental groups, in level 2

**Figure 5:**
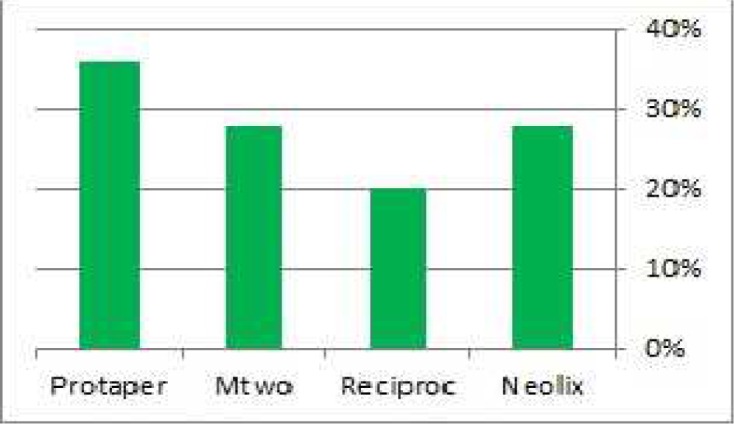
Percentage of dentinal cracks formation found in experimental groups, in level 3

In level 1 the highest and lowest number of cracks were respectively found in root canals prepared with ProTaper (32%) and Mtwo (8%) (Figure 3). In level 2 the highest and lowest one were respectively found in root canals prepared with Reciproc (52%) and Neolix (12%) (Figure 4). In level 3 the highest and lowest one were respectively found in root canals prepared with ProTaper (36%) and Reciproc (20%) (Figure 5). The *Chi*-square test was used to compare the experimental groups in terms of the frequency of cracks in each level and results showed that there was statically no significant difference in this regard among groups except in level 2 (*P*=0.016).

The highest number of cracks were found in level 2 (34%) followed by level 3 (28%) and level 1 (21%). The *Chi*-square test was used to compare the three levels in terms of the frequency of cracks and the results showed that there was significant difference in this regard among the levels (*P*=0.011).

The GEE test was applied for assessments of interaction between groups and levels in terms of the frequency of cracks, which showed no significant difference (*P*=0.249).

## Discussion

Adequate cleaning and shaping of the root canal system is one of important steps in root canal treatment [20]. NiTi rotary files are increasingly used due to less fatigue of the clinician and saving time. On the other hand, risk of crack formation in the root canal walls is a drawback of these systems [21, 22]. 

Conventional rotary systems such as ProTaper have a specific order for use of files at a particular torque and speed specified by the manufacturer [17]. 

Mtwo is a newer rotary system with confirmed advantages over other rotary systems. Its design leads to more flexibility and improved performance [23].

Single-file systems such as, WaveOne, Reciproc, Neolix and OneShape are recently introduced to the market [23]. It is claimed that Neolix and Reciproc systems are well capable of cleaning the root canals with anatomical variations and that the alloy used in these systems enables high flexibility of these files and results in superior adaptation of files to the root canal walls [1, 15]. 

Given that the single-file systems can show equal or superior root canal cleaning efficacy and less damage to root canal walls compared to multi-file systems, they are highly preferred to multi-file systems since they can save time and cost. However, crack formation in the root canal walls is a concern in use of rotary systems, which must be thoroughly evaluated since it can lead to VRF and adversely affect the prognosis [24]. Several studies have evaluated the stress applied to dentin and micro-crack formation in use of rotary systems [4, 21, 22, 25]. However, studies on Neolix, Reciproc and Mtwo are limited. Thus, the current study assessed and compared dentinal crack formation following the use of these systems. The results showed no crack formation in the control groups. Absence of cracks in the unprepared control group ensured that root dentin sectioning did not cause any cracks. This finding has also been confirmed in previous studies [26, 27]. No cracks were noted in the hand file control group either, which was also in accordance with the previous findings [14, 15, 26]. 

Our study showed that there was significant difference in incidence of crack formation in different levels of the root. The highest number of cracks was found in level 2 and the lowest one was in apical third of the root. In the apical third of root, lowest incidence of crack formation was related to Mtwo. Conservative apical third canal preparation is critical for fracture resistance [28], so Mtwo rotary system can be helpful. Our study showed that in midroot (3-6 mm from apex), the highest number of cracks was found with Reciproc and lowest one was found with Neolix. In coronal third of the root, the highest number of cracks was found in root canals prepared with ProTaper and the lowest one was found with Reciproc. This results can related to the differences in taper, flexibility, cross section and other properties of files. There was no data about this subject in literature. 

The current results showed that frequency of cracks formation between experimental groups and control groups statistically significant difference. These findings were in agreement with the results of Kansal *et al.* [29] in 2013, Capar *et al.* in 2014 [30] and Liu *et al.* in 2013 [16]. Kansal *et al.* [29] compared dentinal damage caused by root canal preparation with reciprocating and rotary files with a methodology similar to ours. In their study the highest frequency of cracks was noted in ProTaper and the lowest in WaveOne group. They found a significant difference in this regard between reciprocating and rotary files. Similarly, ProTaper caused the highest frequency of cracks in our study as well. However, in comparison of Neolix and Reciproc systems (with continuous rotation and reciprocal motions, respectively), our results were in contrast to those of Kansal *et al. *[29]; since in their study, the frequency of cracks was lower in WaveOne and Single F2 ProTaper with reciprocating motions than in ProTaper with conventional rotary motion; while in our study, Neolix (with continuous rotation) caused fewer cracks than Reciproc. This controversy may be due to the fact that Neolix has completely different manufacturing process from other NiTi rotary systems and it confers very high flexibility with high microhardness. Combination of these characteristics with a rectangular-shaped cross-section and cutting blades results in high cutting efficacy [31]. Due to increased flexibility, less stress is applied to dentinal walls during rotation of file in the root canal; resultantly, risk of crack formation decreases. Kim *et al.* [32] in 2013 confirmed that the new rotary systems with a modified design and alloy composition apply less stress to root dentin compared to older systems such as ProTaper and thus, it is expected to create fewer cracks in dentinal walls. Capar *et al.* [28] in 2014 also reported that ProTaper caused the highest number of cracks than Hyflex and ProTaper Next. It may be attributed to the fact that the tip of ProTaper finishing files has greater taper than ProTaper Next and Hyflex files. It should be noted that in our study, rotary and Reciproc files were the same in terms of taper and the final file. Thus, differences in the frequency of cracks among different groups cannot be attributed to the taper of files. This difference in the frequency of cracks between experimental groups may be attributed to the design of the file tip, variable or constant taper of rotary file, geometrical shape of the cross-section of the file and flute shape, which are all related to crack formation in root canal walls [24]. Also, the ProTaper Next and Hyflex files, similar to Reciproc, are made of M-Wire NiTi. This alloy has higher cyclic fatigue resistance and greater flexibility than traditional NiTi, which may explain fewer crack formation in M-Wire NiTi compared to conventional NiTi files [29]. In our study, similar to that of Capar *et al.* [28] the M-Wire NiTi file (Reciproc) caused fewer dentinal cracks than ProTaper. Liu *et al.* [16], Yoldas *et al. *[24] and Bier *et al.* [27] reported results similar to ours. However, Burklein *et al. *[33] in 2013 showed that reciprocal files caused more cracks than sequential rotary files, which was in contrast to our findings. This controversy may be due to the fact that Burklein *et al.* [33] did not standardize the teeth in terms of apical diameter. Moreover, the Reciproc file used in their study had different tip and taper to the Reciproc file used in our study and it is probably responsible for the variability in the results of the two studies. 

Jalili *et al. *[15] in 2015 showed that Mtwo caused significantly more cracks than Reciproc, which was in contrast to our findings. Using different tapers of Mtwo rotary files is probably responsible for the variability in the results of the two studies. Topcuoglu *et al.* [34] showed that Neolix caused statistically significant less number of cracks than ProTaper. However, the difference between Neolix and Reciproc was not significant. Search of the literature yielded no previous study on Neolix in this respect. Similar to our findings, Kim *et al.* [32] and Yoldas *et al.* [24] showed that the new rotary systems such as the self-adjusting files apply less stress to root dentin due to different design and alloy composition. Similarly, fewer cracks formed by Neolix in our study may be attributed to its design and different alloy composition from that of ProTaper. Moreover, Neolix is a single file system and application of less stress to dentinal walls with the use of only one file (compared to multiple files) is logical. Bier *et al.* [27] in 2009 indicated that the higher rotation of files in the canal creates the higher number of dentinal crack formation. Thus, it is logical that use of fewer rotary files in the canal results in fewer rotations and subsequently lower number of cracks.

Our study showed that there was statically significant lower frequency of cracks only between Neolix and Mtwo with ProTaper. In contrast to other NiTi files such as ProTaper, Neolix file can be pre-curved and it has a unique way of production. It can be the reason for this difference [14]. 

It should be noted that this study had an *in vitro* design. Thus, generalization of results to the clinical setting must be done with caution. Future clinical studies are required to obtain more reliable results. 

## Conclusion

All rotary systems created dentinal cracks in root canals. There was a significant difference in crack formation in apical, middle and coronal third of the root by all rotary systems. Overall, Neolix caused the least number of cracks with a significant difference with Mtwo and ProTaper. There was a significant difference between rotary systems in crack formation in middle third of the root that Neolix caused the least number of cracks. Thus, it appears that Neolix is a suitable alternative to other rotary systems since use of this single-file system saves time and cost and minimizes trauma to dentinal walls. 
